# Advancing Analysis of Spatio-Temporal Variations of Soil Nutrients in the Water Level Fluctuation Zone of China’s Three Gorges Reservoir Using Self-Organizing Map

**DOI:** 10.1371/journal.pone.0121210

**Published:** 2015-03-19

**Authors:** Chen Ye, Siyue Li, Yuyi Yang, Xiao Shu, Jiaquan Zhang, Quanfa Zhang

**Affiliations:** 1 Key Laboratory of Aquatic Botany and Watershed Ecology, Wuhan Botanical Garden, Chinese Academy of Sciences, Wuhan, 430074, China; 2 College of Environmental Science and Engineering, Hubei Polytechnic University, Huangshi, 435003, China; CAS, CHINA

## Abstract

The ~350 km^2^ water level fluctuation zone (WLFZ) in the Three Gorges Reservoir (TGR) of China, situated at the intersection of terrestrial and aquatic ecosystems, experiences a great hydrological change with prolonged winter inundation. Soil samples were collected in 12 sites pre- (September 2008) and post submergence (June 2009) in the WLFZ and analyzed for soil nutrients. Self-organizing map (SOM) and statistical analysis including multi-way ANOVA, paired-T test, and stepwise least squares multiple regression were employed to determine the spatio-temporal variations of soil nutrients in relation to submergence, and their correlations with soil physical characteristics. Results showed significant spatial variability in nutrients along ~600 km long shoreline of the TGR before and after submergence. There were higher contents of organic matter, total nitrogen (TN), and nitrate (NO3-) in the lower reach and total phosphorus (TP) in the upper reach that were primarily due to the spatial variations in soil particle size composition and anthropogenic activities. Submergence enhanced soil available potassium (K), while significantly decreased soil N, possibly due to the alterations of soil particle size composition and increase in soil pH. In addition, SOM analysis determined important roles of soil pH value, bulk density, soil particle size (i.e., silt and sand) and nutrients (TP, TK, and AK) on the spatial and temporal variations in soil quality. Our results suggest that urban sewage and agricultural runoffs are primary pollutants that affect soil nutrients in the WLFZ of TGR.

## Introduction

Nutrient pollution in a watershed has a large variety of sources such as industrial and domestic wastewater, agricultural runoff, mining activities, and atmospheric deposition [1]. Excessive loads of certain nutrients often cause eutrophication, water quality deterioration, and biodiversity loss in aquatic ecosystems [2]. Riparian wetlands, located at the intersection of terrestrial and aquatic ecosystems, play an important role in mediating nutrient pollution for rivers [3], [4]. Therefore, it is essential to characterize nutrient dynamics in the riparian zone for an improved assessment of environmental quality.

Nutrient levels in riparian zone depend on a variety of factors, such as soil and vegetation characteristics, flooding, and anthropogenic activities [5], [6]. Soil characteristics particularly soil pH, soil particle size composition, and redox condition have large effects on the sorption-desorption processes and microbial activities, and thus regulate soil nutrient dynamics [7–9]. For instance, Brunet and Astin [10] found that coarser sandy sediment was associated with low level of organic material and a mixture of silt and clay being associated with enhanced concentrations of organic matter and soil nutrient. Riparian vegetation could regulate soil nutrient contents directly by uptake, decomposition and deposition of alluvial materials, and indirectly through stimulation of microbial processes in the rhizosphere [11–13]. The input of organic matter by plant could generate high rates of microbial activity [14], [15]. Vegetation decomposition contributes to short-term N retention in riparian zones by consuming nutrients from external sources during the first stages of the decomposition [12]. Therefore, Nitrate would be removed more effectively in densely vegetated riparian zones due to the plant uptake and high rates of nitrification and denitrification [2], [12], [13], [16].

Flooding is also an important factor regulating nutrient levels in riparian areas [9], [17–19]. Seasonal drawdown and exposure poses alternative aerobic and anaerobic conditions which have important influences on soil mineralization and nutrient cycling in riparian zones [20], [21]. Submergence creates anaerobic soil environment, which transforms nitrate to nitrogen (N_2_), nitrous oxide (NO_2_), and/or ammonium (NH_3_) and increases P availability due to the reduction and dissolution of iron phosphate [22], [23]. In reservoir system, nutrients sedimentation and subsequent sediment-water interactions are primary regulatory processes affecting the nutrient status in the riparian zone during the submergence [23], [24]. Moreover, anthropogenic activities, i.e., the mass use of fertilizer and pesticide in the uplands release large quantity of nitrates and phosphates, and understandably increase their concentrations in the riparian zone [25], [26].

Traditional statistical methods, i.e., principal component analysis, hierarchical cluster analysis, and correlation, have been widely applied to investigate the relationship between soil nutrients and various influence factors [27], and the results were always based on various sources of statistical bias except that all the requirements in the analysis were strictly adjusted [28]. Thus, it is inherently difficult to assess and understand soil nutrient dynamics and their associated controls. The Kohonen Self-Organizing Map (SOM) [29], an artificial neural network has been applied as an alternative to conventional multivariate statistical approaches in environmental data [30]. SOM could reveal different effects of nutrient pollutions and spatio-temporal variations in environmental conditions [31].

Dams have profound influences on the environments and ecological processes of riverine systems and beyond [32–36]. After the completion of the Three Gorges Dam in 2008, water level fluctuated from 145 m in summer (May to September) to 175 m in winter (October to April), and a total area of 350 km^2^ water level fluctuation zone (WLFZ) was formed in the Three Gorges Reservoir [37]. The reversal of submergence time and prolonged inundation result in loss of previous vegetation which would have influenced soil nutrient dynamics in the riparian ecosystem. Moreover, 1.2×10^9^ tons of industrial and domestic sewage were discharged into the Reservoir in 2008, of which ~80% of the sewage had received some level of treatment before discharge [38]. In addition, intensive agriculture activities in the middle and low regions of the Reservoir caused around 1.4×10^5^ tons of chemical fertilizers and 5.3×10^2^ tons of pesticide used for agriculture (a total of 977,700 ha) in 2008, of which ~9% was lost by surface runoff [38]. Therefore, anthropogenic impacts on the soil quality in the WLFZ are of great public interest and concern. In this study, multi-statistical approaches and SOM were used to examine the spatio-temporal variability of soil nutrients and their associated dominant controls before and after submergence in the WLFZ. Our results would help understand the great impacts of submergence on soil nutrients and develop strategies for revegetation in the WLFZ.

## Materials and Methods

### 2.1 Site description

**Fig 1:**
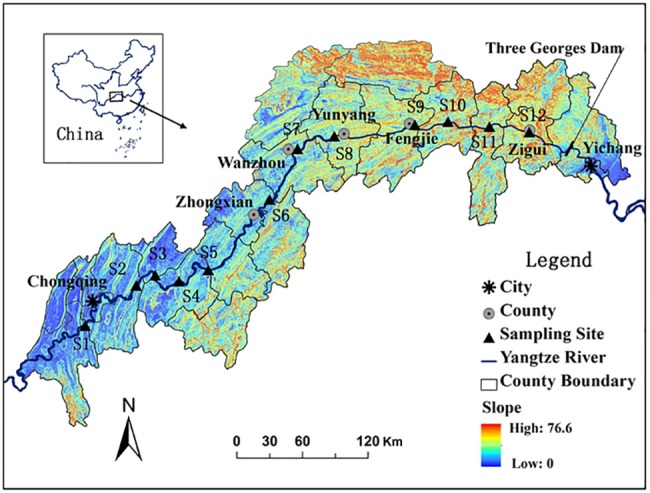
Location of the sampling sites in the water level fluctuation zone of the Three Gorges Reservoir, China (upper reach includes sites 1–4, middle reach includes sites 5–8, and lower reach includes sites 9–12). The original image was cited from Ye et al. [52] with differences in sampling sites.

The Three Gorges Reservoir region (29°16′- 31°25′ N, 106°- 111°50′ E) lies in a 600 km valley from Yichang to upstream Chongqing (Fig. 1). Climate in this region belongs to southeast sub-tropic monsoon. Annual mean temperature is 16.5–19°C with the highest and lowest temperatures of 28°C and 3.4°C, respectively. Annual precipitation is about 1100 mm with 80% falling from April to October. Zonal soil types include red soil, yellow soil, and mountain yellow soil [39]. With the Three Gorges Dam fully functioning in 2008, the water level of the reservoir fluctuates from 145 m *a*.*s*.*l* in summer (May to September) to 175 m *a*.*s*.*l* in winter (October to April) thereafter. The reversal of flooding time and prolonged submergence have dramatically altered the hydrological regime in the WLFZ [40].

### 2.2 Vegetation in the WLFZ

Before submergence (September 2008), vegetation in the 145–155 m were dominated by annuals, *i*.*e*., *Setaria viridis*, *Digitaria ciliaris*, and *Leptochloa Chinensis*, and perennials including *Cynodo dactylon*, *Hemarthria altissima*, and *Capillipedium Assimile*, and from 155–175 m vegetation were dominated by woody plants such as *Ficus tikoua*, *Pterocarya stenoptera*, and *Vitex negundo* in the WLFZ [41]. After submergence (June 2009), the vegetation in the WLFZ was uniformly composed of annual and perennial herbs such as *S*. *viridis*, *D*. *ciliaris*, and *C*. *dactylon* [42].

### 2.3 Soil sampling

Field surveys were conducted in September 2008 (before submergence) and June 2009 (one week after submergence) when the reservoir’s water level in 145 m and the WLFZ was exposed to the air. Twelve sampling sites were selected based on the geographical characteristics of the WLFZ from upstream to downstream in the Three Gorges Reservoir (Fig. 1) and divided into the upper reach (Sites 1–4), middle reach (Sites 5–8), and lower reach (Sites 9–12). Each sampling location was allowed by the Executive Office of the State Council Three Gorges Construction Committee. Land use history in these sites was deserted factories or croplands abandoned for more than 5 years and the major soil type was yellow soil. Each sampling site was marked by PVC pipes and measured by GPS to ensure soil sampling on the same site before and after submergence.

At each sampling site, six plots (1 m × 1 m each) were randomly selected in the three intervals of elevation from 145 to 175 m (i.e., 145–155 m, 155–165 m, and 165–175 m) in order to examine the influence of flooding depth and duration on soil nutrients. At each plot, five replicates from 0–20 cm layer soil were selected evenly throughout the plot and well mixed to form a composite sample. However, only two samples were collected in site 1 between the elevations of 165–175 m because of the high water level during the sampling period. Thus, a total of 136 samples were collected, and all the samples were sealed in plastic bags and stored at 4°C for analysis.

### 2.4 Soil sampling analysis

Soil organic matter (OM) was determined by potassium bichromate (K_2_Cr_2_O_7_) titration solution after digestion. Total nitrogen (TN) was measured using a nitrogen/carbon analyzer (NA-1500-NC Series 2) with Eager 200 software (Fisons Instruments, Beverly, MA, USA). For total phosphorus (TP) and total potassium (TK) analysis, soil digestion was performed in Kjeldahl flask following the classical open digestion procedures with a mixture of concentrated HClO_4_-H_2_SO_4_ (i.e., 1ml HClO_4_ and 10ml H_2_SO_4_). TP was determined by molybdenum blue colorimetry method; TK was measured by flame photometry method; Available phosphorus (AP) was determined by 0.5M NaHCO_3_ extraction (1:20) colorimetric method; and available potassium (AK) was determined by 1M NH_4_OAC extraction (1:20) flame photometry method [39]. The ammonium (NH_4_^+^) and nitrate (NO_3_^-^) contents were measured by extraction of 20 g of fresh soil with 100 ml extractant (i.e., 0.4 M KCl) for 1 h. After filtering the suspension, the extract was analyzed for NH_4_^+^ and NO_3_^-^ on a continuous flow auto analyzer (Skalar-40) using a colorrmetric method [43]. Soil pH was measured in a 2:1 (by weight) soil to water solution using Fisher Scientific AR15 (Waltham, MA) pH probe. Soil water content was measured by dried at 105°C. The particle-size was determined by wet sieving and by sedimentation using the pipette sampling technique [44]. The relative elevation above the water level was estimated by the absolute elevation subtracting the low water level (145 m) of the reservoir. The average flooding duration (sum of days per year) was derived from daily water level data (http://xxfb.hydroinfo.gov.cn/).

### 2.5 Self-Organizing Map and statistical analysis

SOM provides projection of multidimensional matrix patterns into a two-dimensional map preserving the topology of the input data based on an unsupervised Kohonen’s learning algorithm [27]. SOM consists of one hexagonal grid formed by units called nodes, and a weight vector with the same dimensions as the number of input variables, associated with each node [45]. The main algorithms in the SOM are topology conserving mapping and vector quantization. Four different processes including vector initialization, competition, cooperation, and adaptive processes are implemented to eventually determine the coordinates for each corresponding observation in the map. More details and guidance regarding the SOM are provided in literature [29], [46].

In this study, input data vectors consisted of 7 soil nutrient variables and 9 relevant parameters (i.e., soil particle size composition, relative elevation, flooding duration, pH, bulk density, soil moisture and organic matter). Each data vector component was normalized in a scale from 0 to 1 after range scaled transformation to avoid the potential effect of magnitude difference among data vectors on the final map determination. The number of nodes was determined based on the number of samples or 5$\sqrt{n}$ to guarantee a small error [27], [45]. Here, SOM was trained with 72 nodes for each sampling times, corresponding to the 68 samples, and 56 nodes (≈5$\sqrt{136}$) for the total samples. Samples within the same node would be the most similar in terms of the variables considered, while more different samples were expected to be distant in the map.

In order to explain the results of the SOM, component planes were derived by taking the same component of the weight vectors in each of the map node. There are as many component planes as data variables (here 15 variables for before-submergence and 16 for after-submergence). The component plane provides an idea of the spread for a particular variable, and correlations between variables can be seen by comparing component planes (so that similar distributions show positive correlations while inverse distributions indicate negative correlations) [30]. SOM analyses were performed using MATLAB 7.9 for Microsoft Windows.

Multi-way ANOVA was performed to investigate the spatial variations in soil nutrients and characteristics after the normal distribution test and homogeneity test of variance. Least significant difference (LSD) test was used for the identification of the groups which differed significantly and Dunnett’s C test was also performed to analyze the differences when the parameters did not meet the homogeneity test of variance even by transformation. Paired-T test were performed to investigate the effects of sampling time on soil nutrients and characteristics. Stepwise least squares multiple regression with soil nutrient variables as dependent variables was carried out to assess relations among soil characteristics. All the processes were performed using SPSS 13.0 for Microsoft Windows.

## Results

### 3.1. Soil nutrients variations

**Fig 2:**
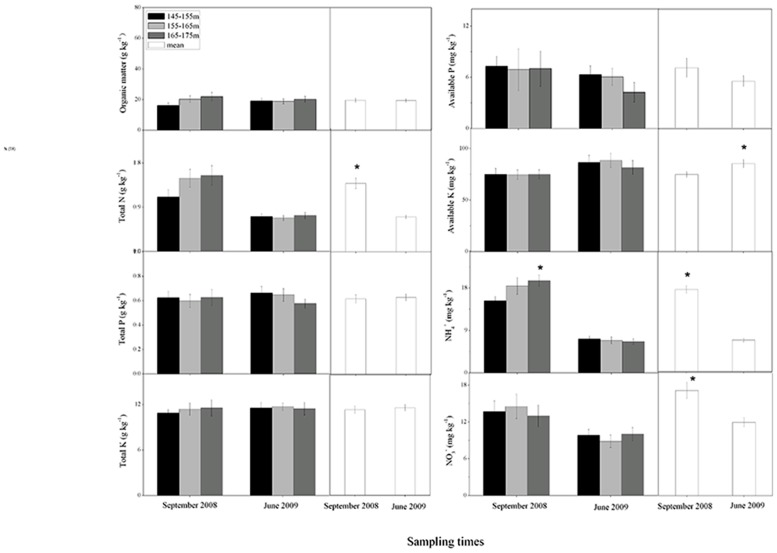
Soil nutrients at three elevation intervals and annual averages before submergence (September 2008) and after submergence (June 2009). Note: asterisk (*) indicating significant differences at α = 0.05 level between elevation intervals for each sampling time and between annual averages.

All nutrients except AK showed significant spatial variations among the upper, middle and lower reaches (p < 0.05 by ANOVA) with higher contents of OM, TN, and NO_3_^-^ in the lower reach (Site 1–4), and TP in the upper reach both before and after submergence (Table 1). There were no significant differences in soil nutrients among the three elevational intervals (i.e., 145–155, 155–165, 165–175) during the two sampling times, except for NH_4_^+^ with the highest level in the elevation of 165–175 m before submergence (Fig. 2). The concentration of AK significantly increased, while the levels of TN, NH_4_^+^, and NO_3_^-^ decreased after the inundation (p < 0.001) (Fig. 2).

**Table 1:** Characteristics of soil nutrients before submergence (September 2008) and after submergence (June 2009) in the upper, middle and lower reaches of the water level fluctuation zone of the Three Gorges Reservoir (mean±SE).

	Before-submergence			After-submergence		
	Upper (n = 20)	Middle(n = 24)	Lower (n = 24)	Upper (n = 20)	Middle (n = 24)	Lower (n = 24)
pH	7.45±0.07b	7.22±0.15b	7.74±0.05a	8.26±0.03a	7.88±0.16a	8.03±0.12b
Bulk density (g cm^-3^)	1.65±0.05ab	1.71±0.04a	1.58±0.04b	1.39±0.04a	1.47±0.04a	1.49±0.03a
Soil water content (%)	23.13±2.38a	17.20±1.19a	21.10±1.13a	23.52±2.17a	16.28±1.36b	14.94±0.58b
Organic matter (g kg^-1^)	13.59±1.31b	16.18±1.79b	27.11±2.22a	16.65±1.15b	16.72±1.05b	23.93±2.27a
Total N (g kg^-1^)	0.78±0.09c	1.33±0.15b	1.88±0.19a	0.57±0.03b	0.59±0.04b	0.93±0.06a
Total P (g kg^-1^)	0.71±0.02a	0.70±0.07a	0.47±0.03b	0.82±0.04a	0.64±0.04b	0.48±0.03c
Total K (g kg^-1^)	10.17±0.65a	12.78±1.12a	10.60±0.29a	11.69±0.68ab	12.83±0.81a	10.23±0.42b
Available P (mg kg^-1^)	6.10±0.87a	10.55±2.81a	4.28±0.43a	6.70±1.15a	6.69±1.32a	3.46±0.39b
Available K (mg kg^-1^)	81.53±6.39a	70.68±2.73a	74.46±5.33a	74.94±4.46a	87.50±6.11a	90.95±7.75a
NH_4_^+^ (mg kg^-1^)	18.14±0.80ab	19.82±1.59a	15.58±1.19b	5.92±0.28a	6.94±0.45a	7.53±0.82a
NO_3_^-^ (mg kg^-1^)	8.54±0.98b	14.67±1.47a	16.44±2.10a	7.92±1.51b	9.06±0.66ab	11.28±0.88a

Significant differences at α = 0.05 level (multi-way ANOVA) are indicated by different letters.

### 3.2 Soil granulometric variations

**Fig 3:**
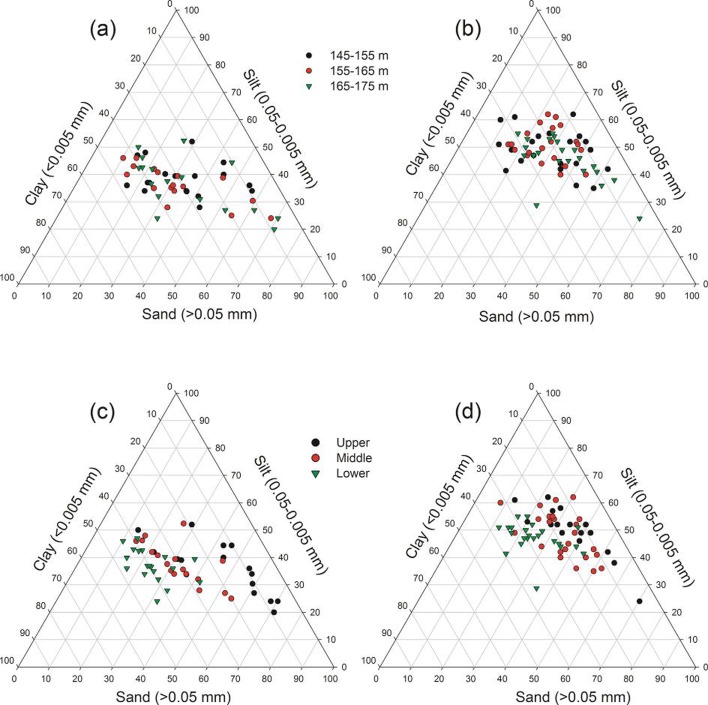
Spatial variations on soil particle size composition before submergence (September 2008) (a and c) and after submergence (June 2009) (b and d) in the water level fluctuation zone of the Three Gorges Reservoir.

The soil particle size excepting silt showed significant spatial variation among the upper, middle and lower reaches (p < 0.05) with higher contents of sand in the upper reach and clay in the lower reach both before and after submergence (Fig. 3a, 3b). There were no significant differences in soil particle size among the three elevational intervals (p > 0.05) before submergence, while the soil particle size in the elevation of 155–165 m altered significantly with higher silt and lower sand contents after submergence (Fig. 3c, 3d). The silt content and soil pH significantly increased, and clay and bulk density decreased after submergence (p < 0.001) (Fig. 3; Table 2).

**Table 2:** Environmental factors before submergence (September 2008) and after submergence (June 2009) (mean±SE) at three elevation intervals (i.e., 145–155m, 155–165, 165–175m).

	pH	Bulk density (g cm^-3^)	Soil water content (%)	Relative elevation (m)	Flooding duration (day)
Before submergence					
145–155 m (n = 22)	7.65±0.05a	1.69±0.04a	21.46±1.93a	3.29±0.91c	
155–165 m (n = 22)	7.43±0.12a	1.64±0.05a	19.87±1.28a	15.29±0.78b	
165–175 m (n = 24)	7.34±0.14a	1.60±0.05a	19.33±1.47a	23.57±1.57a	
Mean (n = 68)	7.47±0.07B	1.65±0.03A	20.19±0.91A		
After submergence					
145–155 m (n = 22)	8.15±0.04a	1.44±0.04a	18.14±1.44a	3.29±0.91c	261±7a
155–165 m (n = 22)	7.95±0.16a	1.45±0.03a	18.65±1.84a	15.29±0.78b	146±5b
165–175 m (n = 24)	8.02±0.15a	1.49±0.04a	16.37±1.37a	23.57±1.57a	79±14c
Mean (n = 68)	8.04±0.07A	1.46±0.02B	17.68±0.89A		

Lowercase letters (a, b and c) demonstrate the effects of elevation on soil characteristics with significances at α = 0.05 level (tested by Multi-way ANOVA), uppercase letters (A and B) represent the effect of sampling time on soil characteristics with significant differences at α = 0.05 level (reflected by Paired-T test).

### 3.3 Relationship between soil nutrients and other relevant parameters

**Fig 4:**
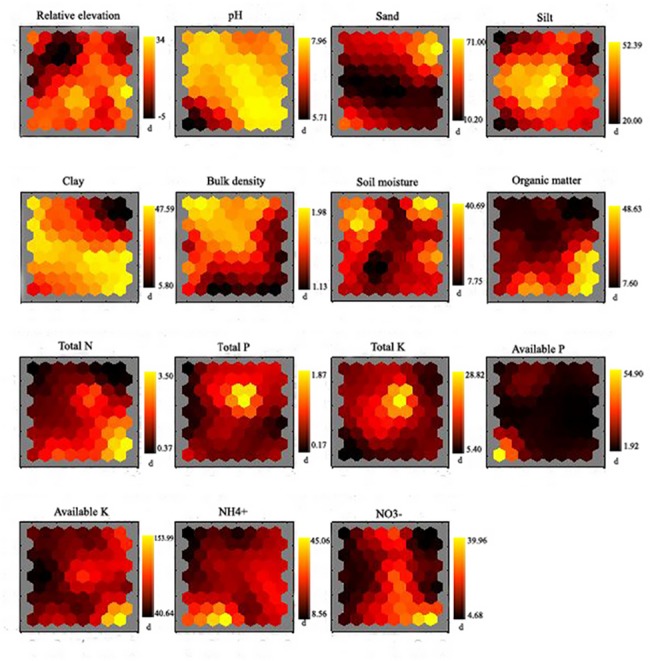
Relationship among concentration of soil parameters, and relative elevation by the visualization of component planes in a SOM before submergence. Vertical bars in the jet colormap present the measure values, i.e., relative elevation in (m), bulk density in (g cm^-3^), soil moisture and particle size composition in (%), total nutrients in (g kg^-1^), and available nutrients in (mg kg^-1^).

**Fig 5:**
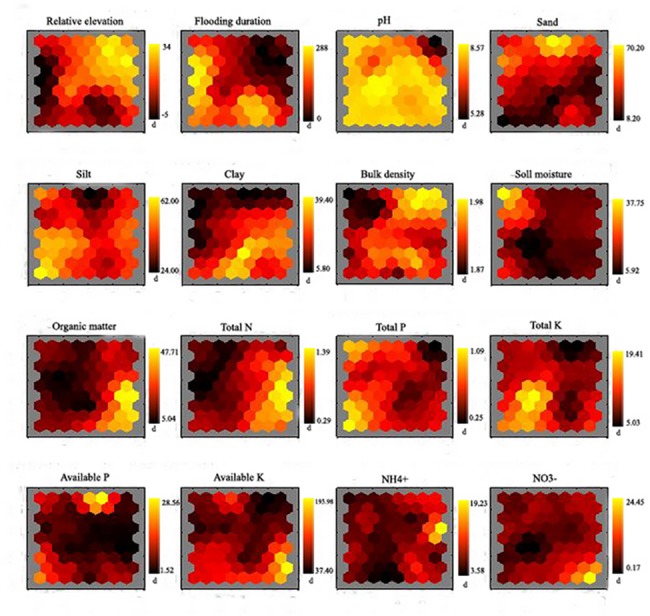
Relationship among concentration of soil parameters, relative elevation, and flooding duration by the visualization of component planes in a SOM after submergence. Vertical bars in the jet color map present the measure values, i.e., relative elevation in (m), flooding duration in (d), bulk density in (g cm^-3^), soil moisture and particle size composition in (%), total nutrients in (g kg^-1^), and available nutrients in (mg kg^-1^).

Regardless the submergence, OM was strongly positively correlated with TN, AK and NO_3_^-^, sand negatively with TK, silt positively with TP and TK, and clay positively with OM and TN based on the SOM analysis (Figs. 4, 5). Before submergence, pH and BD were negatively related to AP and OM, respectively, and TP was positively related to TK (Fig. 4). After submergence, sand was negatively associated with TP and TK, pH was negatively associated with NH_4_^+^, and relative elevation was negatively with TP and TK (Fig. 5).

Stepwise multiple linear regression indicated that TN was predicable by OM both before and after submergence. Soil physical property was a very important predictor for soil nutrient contents, before submergence, while after submergence, only silt was one of the factors for TK. pH was also a very important predictor for soil nutrients (Table 3).

**Table 3:** Stepwise multiple regression models for soil nutrients before submergence (September 2008) and after submergence (June 2009) in the water level fluctuation zone of the Three Gorges Reservoir, China.

	Independent variables	Regression equations	R^2^	Adjusted R^2^
Before submergence				
Total N (TN)	Organic matter (OM)	0.140 + 0.064 OM	0.654^b^	0.648^b^
Total P (TP)	TK and Clay	0.462 + 0.047 TK-0.014 Clay	0.670^b^	0.659^b^
Total K (TK)	TP and Sand	9.712 + 10.516 TP—0.148 Sand	0.631^b^	0.619^b^
Avaliable P (AP)	pH	79.051–9.639 pH	0.331^b^	0.321^b^
Available K (AK)	TN and Clay	76.267 + 14.192 TN—0.706 Clay	0.211^a^	0.186^a^
NH_4_^+^	OM and pH	48.924 + 0.293 OM—4.933 pH	0.429^b^	0.410^b^
NO_3_^-^	TN	7.185 + 4.709 TN	0.220^b^	0.207^b^
After submergence				
TN	OM	0.251 + 0.024 OM	0.538^b^	0.531^b^
TP	pH and AP	-0.933 + 0.180 pH + 0.021 AP	0.455^b^	0.438^b^
TK	Silt, OM and AK	2.009 + 0.174 Silt—0.175 OM + 0.053 AK	0.480^b^	0.455^b^
AP	TP	-1.089 + 10.481 TP	0.210^b^	0.197^b^
AK	OM and TK	-8.092 + 2.023 OM + 4.702 TK	0.419^b^	0.400^b^
NH_4_^+^	TN and pH	20.305 + 3.302 TN—1.961 pH	0.252^b^	0.228^b^
NO_3_^-^	OM	5.084 + 0.232 OM	0.175^a^	0.162^a^

Note: Sand: soil particle size greater than 0.05 mm; Silt: soil particle size between 0.05 and 0.005mm; Clay: soil particle size smaller than 0.005 mm;

^a^ Significance at the 0.01 probability level;

^b^ Significance at the 0.001 probability level.

### 3.4 Parameters dominating soil quality variation

**Fig 6:**
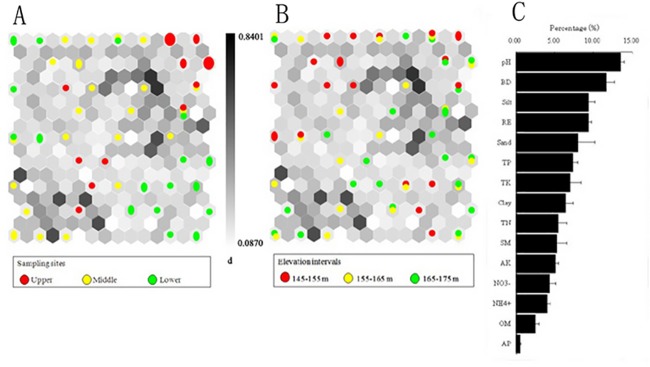
Spatial difference of soil quality parameters before submergence: (A) hit histogram (i.e., data density indicated by the circle size) in a U-matrix illustrating the differences among sampling sites (upper-red circles, middle-yellow circles, and lower-green circles), (B) hit histogram indicating variations among three elevation intervals (145–155 m-red circles, 155–165 m-yellow circles, and 165–175 m-green circles), and (C) rank of significant parameters from the highest rank in the top to the lowest in the bottom. A larger circle denotes a higher density of the data in the hexagonal grid unit, and the grayscale is the U-matrix representing the Euclidean distance between the neighboring map units (i.e., grayscale located at the intermediate position between the units). Light regions imply a high degree of similarity between the units, whereas dark areas represent a large distance. Percentage statistics (means and standard error) for the rank are averaged based on the first four SOM units with larger Euclidean distance among data vectors (Ki et al., 2011).

**Fig 7:**
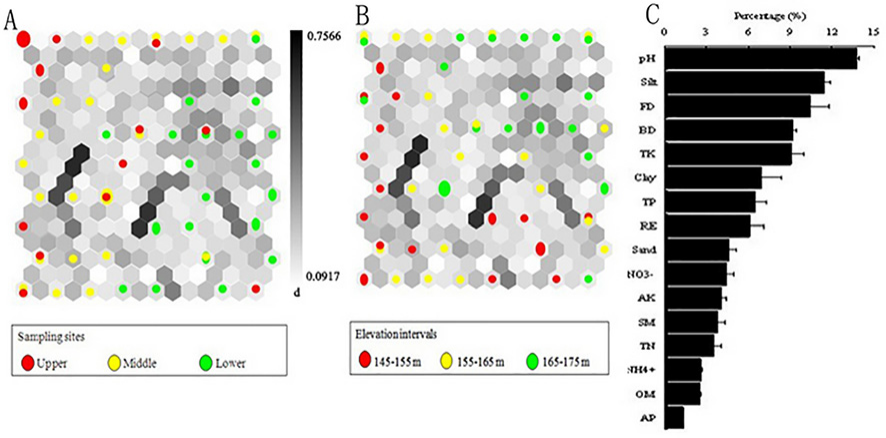
Spatial difference of soil quality parameters after submergence: (A) hit histogram (i.e., data density indicated by the circle size) in a U-matrix for presenting differences among sampling sites including upper (red circles), middle (yellow circles) and lower (green circles), (B) hit histogram for indicating variations among three elevation intervals, i.e., 145–155 m (red circles), 155–165 m (yellow circles) and 165–175 m (green circles), and (C) rank of significant parameters from the highest rank in the top to the lowest in the bottom. Symbols are similar to Fig. 6.

**Fig 8:**
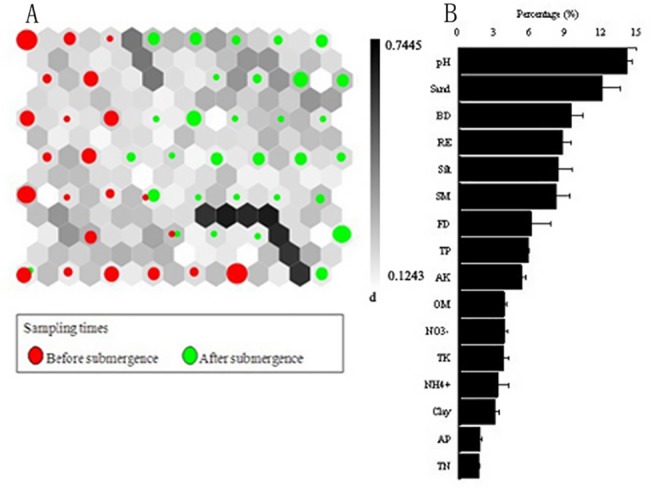
Temporal variations of soil quality parameters: (A) hit histogram (i.e., data density indicated by the circle size) in a U-matrix for presenting differences between before-submergence (red circles) and after-submergence (green circles), and (B) rank of significant parameters from the highest rank in the top to the lowest in the bottom. Symbols are similar to Fig. 6.

Figs. 6 and 7 illustrated the spatial variabilities of soil quality among the upper (red circles), middle (yellow circles) and lower reaches (green circles) in the WLFZ and their dominant influencing factors. Before submergence, the samples corresponding to the upper and lower reaches were concentrated in a more compact region in the map than those in the middle reach (Fig. 6A). Samples belonging to each elevation interval were evenly distributed (Fig. 6B). The pH and bulk density had significant effects on the boundaries constructed, and TP and TK had relatively more important effects on soil quality compared to other soil nutrients (Fig. 6C). After the submergence, samples belonging to each cluster were evenly distributed across the SOM map (Fig. 7A), while samples in the elevation of 145–155 m were concentrated in a more compact region than samples in other elevations (Fig. 7B). The pH and silt showed significant effects on the boundaries and were associated with TK and TP, while AP, OM, NH_4_^+^ and TN had lower impacts on the borders (Fig. 7C). Fig. 8 depicted the temporal variability of soil quality before submergence (red circles) and after submergence (green circles) in the WLFZ. The pH and sand were associated with TP and AK, and played an important role in the boundaries, whereas negligible or smaller effects were observed for TN, AP, clay and NH_4_^+^.

## Discussion

### 4.1 Spatial variations in soil nutrients and dominant influencing factors

The changes of soil characteristics (i.e., soil particle size composition and pH) induced by inundation and anthropogenic activities understandably resulted in significant spatial variability in soil nutrients in the WLFZ (p < 0.05) both before submergence (September 2008) and after submergence (June 2009) (Tables 1 and 3). Similar observations were found elsewhere [2], [8], [25], [39], [47], [48]. Specific discussions are as follows. In the upper reach, turbulent flow would scour smaller soil particles away and leave much coarser sandy sediment with low organic materials (Table 3; Fig. 3,). Conversely, in the lower reach, calm water might have deposited a mixture of silt and clays being associated with enhanced concentrations of OM and TN (Figs. 3, 4, 5) [10]. The spatial variability of soil pH value also contributed to the spatial variations on soil nutrients such as AP and NH_4_^+^ by affecting the nutrient dissolution processes and thereby regulating the soil nutrient availability (Table 3) [6]. In addition, anthropogenic pollutants, i.e., domestic sewages and agricultural runoffs from upland [38], also contributed to higher nutrient contents in the lower reach (Site 9–12). For instance, Site 9 is located in Fengjie County with high population density and intensive agricultural activities which discharges a large amount of wastewater containing high loads of N (Fig. 1; Table 1) [38].

SOM toolbox was applied to assess the degree of influence of individual parameters (i.e., 7 soil nutrients, soil particle size composition, relative elevation, flooding duration, pH, bulk density, soil moisture and organic matter) [27]. Before submergence, intensive anthropogenic activities (i.e., high dense population in the upper reach and intensive agricultural activities in the lower reach) caused the relative concentrating distribution of soil quality in the upper and lower reaches (Fig. 6A), indicating that the soil quality in these regions was less variable.

To better illustrate the dissimilarity in soil quality among the upper, middle and lower reaches, we investigated which parameters played an important role in determining the spatial variation [31]. Before submergence, pH and bulk density had a significant effect on the boundaries constructed by SOM, while after submergence pH and silt played an important role in the borders (Figs. 6C, 7C), indicating that the soil particle size composition had been greatly altered during hydrologic processes. Moreover, TP and TK had relatively more important effects on the spatial variability of soil quality compared to other soil nutrients both before and after submergence (Figs. 6C, 7C), which implied that the non-point pollution by agricultural activities, i.e., the usage and discharge of fertilizer and pesticide, had greatly influenced soil quality by altering the contents of soil TP and TK. Compared to the findings obtained by the statistical analysis (i.e., multi-way ANOVA) (Table 1), the parameters were identified by SOM with little or no effect on the borders without measurable difference. However, there was disparity between the two analysis methods, especially for organic matter, TN and NO_3_^-^, which could be attributed to the differences in the number of samples usable for the computation and calculation way (i.e., a reduced number or samples and training algorithm in SOM, and raw data and statistical calculations for the statistical analysis) [27]. Accordingly, these results revealed that the spatial variations on soil quality could be largely explained by soil pH and physical characteristics including soil particle size composition.

### 4.2 Changes in soil nutrients after submergence

Submergence could alter soil nutrients in the riparian zone directly by affecting their physical and chemical processes, including sorption-desorption and solution-dissolution processes and indirectly by altering the vegetation distribution [5], [7]. In the present study, submergence significantly decreased the soil TN, NH_4_^+^, and NO_3_^-^ concentrations and increases the content of soil AK (Fig. 2). The decreases in soil NH_4_^+^ and NO_3_^-^ after the inundation were possibly due to the increase in soil pH after submergence (Table 2 and Fig. 2). The results were in consistent with the findings of Wang et al. [6], who demonstrated that the increased pH could reduce the availability of soil N, leading to the decrease in N species. Furthermore, the increase in AK and decrease in TN were related to the increased silt and decreased clay after inundation (Table 3; Figs. 2 to 5) [7]. Changes of soil granulometric nature could affect the sorption-desorption and soil microbial nitrogen processes (i.e., denitrification) and cause temporal fluctuations of nutrient concentrations [7], [10]. In addition, the periodic submergence could also regulate the soil nutrient dynamics by frequent material exchange between water and riparian zone, which was an equilibrium process of adsorption and release [6]. The WLFZ could absorb a mass of inorganic N from surface runoff in the exposed period, and release more inorganic N into the overlying water during submergence leading to the observed decreased in concentration of N after submergence (Fig. 2) [49]. Therefore, the risk of eutrophication seemed to increase in the Three Gorges Reservoir.

Decreases in plant diversity and richness by inundation also contributed to the temporal dynamics of soil nutrient [10], [11]. Six-month submergence in the WLFZ clears the most of the pre-dam vegetation and a bald zone is formed in the WLFZ before natural rehabilitation [37]. This could promote natural chemical weathering and thus partly contribute to the enhanced soil AK after submergence (Fig. 2) [6]. Moreover, denitrification is viewed as the dominant attenuation process that controls nitrogen fluxes along river systems [7], [15], [50]. Submergence-induced anaerobic soils and increase of organic matter by the decaying litter would promote microbial denitrification [15], [22], [51], leading to the decreased NO_3_^-^ during the submergence period (Fig. 2). Furthermore, vegetation decomposition after submergence may temporarily reduce the amount of inorganic N in the riparian zone (Fig. 2), because at the first stage of decomposition consumes nutrients from external sources [12]. Plant uptake of inorganic nutrients for growth is also an important process that was correlated to the decreases in soil NH_4_^+^, and NO_3_^-^ concentrations after submergence (Fig. 2) [3], [15].

SOM analysis results revealed obviously temporal variations on soil quality after submergence (Fig. 8A). The soil quality in the upper and lower reaches were changed from relative concentrated distribution before submergence to even distribution after submergence (Figs. 6A, 7A), implying that submergence showed large effects on soil quality. Compared to other soil nutrients, pH and sand, associated with TP and AK played an important role in the boundaries constructed by SOM (Fig. 8C), implying that submergence had greatly influenced soil quality by changing soil pH and particle size composition. Our results indicated that the SOM has an additional benefit for describing dynamics of soil quality and identifying potential parameters such as pH and soil particle size characteristics that dominate temporal variation in soil quality. Albeit the inherent disparity between the two approaches exist due to the difference in computational schemes and the number of samples applied.

### 4.3 Impacts of depth and duration of inundation on soil parameters

Soil nutrients except for NH_4_^+^ did not show significant variations among different altitudes after submergence (June 2009) (Fig. 2), which implied little impact of flooding depth and duration on soil nutrients. However, after the submergence, there were significant differences in sand and silt among the elevation intervals (Fig. 3) and samples in the elevations of 145–155 m were concentrated in a more compact region than other samples (Fig. 7B). These results indicated that the flooding depth and duration had some impacts on soil particle size composition. However, it was relative a short time period for the formation of WLFZ and the environment is still in a state of transition. Thus, long-term field studies are necessary to extrapolate the flooding depth and duration effects on soil nutrients in the WLFZ.

### 4.4 Implications

Seeking appropriate approaches to restore and protect the riparian ecosystems will be essential for the sustainable development of the Three Gorges Reservoir and remain a high priority for the country. However, any efforts have to depend on the understandings of the environmental changes in the region. With the resident relocations, urbanization and economic development, pollutants from industrial and domestic wastewaters and agricultural runoffs have exceptionally affected the ecological environment in the reservoir region [40]. Our present study together with the investigation by Zhan et al. [49] highlighted that inorganic N absorbed by the WLFZ from surface runoffs in the exposed period would be largely released from soil into the overlying water during submergence period. Our previous studies also demonstrated that revegetation in the WLFZ could decrease soil inorganic N and potentially improve water quality [15]. Therefore, revegetation could be a sound practice for ecological restoration by planting native species to absorb large quantity of nutrients and reduce soil erosion.

## Conclusion

Soil nutrients excepting AK showed significant spatial variability both before and after submergence in the water level fluctuation zone (WLFZ) of the Three Gorges Reservoir, China. Higher contents of OM, TN, and NO_3_^-^ in the lower reach and TP in the upper reach were closely related with soil particle size composition and anthropogenic pollutants including domestic sewages and agricultural runoffs. The increase in soil AK and decrease in AP, TN, NH_4_^+^, and NO_3_^-^ after submergence resulted from changes of soil particle size composition and increased soil pH that greatly influenced the nutrient sorption-desorption and dissolution processes. SOM visualized the dynamic patterns of soil quality variables and identified potential parameters including pH, bulk density, soil particle size (i.e., silt and sand), and nutrients (TP, TK and AK) as the primary determinants for the spatial and temporal variations of soil quality. Our results provided a clear evidence that riparian ecosystems respond differently to the inundation [22], [26], which in turn provides strategies for revegetation and water quality protection.
